# Functional, size and taxonomic diversity of fish along a depth gradient in the deep sea

**DOI:** 10.7717/peerj.2387

**Published:** 2016-09-15

**Authors:** Beth L. Mindel, Francis C. Neat, Clive N. Trueman, Thomas J. Webb, Julia L. Blanchard

**Affiliations:** 1 Department of Animal and Plant Sciences, University of Sheffield, Sheffield, United Kingdom; 2 Marine Scotland, The Scottish Government, Aberdeen, United Kingdom; 3 Ocean and Earth Science, University of Southampton, Southampton, United Kingdom; 4 Institute for Marine and Antarctic Studies, University of Tasmania, Hobart, Australia

**Keywords:** Bathymetry, Deep scattering layer, Environmental gradient, Morphometrics, Rockall Trough, Trait-based approach, Demersal fish, Continental slope, Biodiversity, Functional role

## Abstract

Biodiversity is well studied in ecology and the concept has been developed to include traits of species, rather than solely taxonomy, to better reflect the functional diversity of a system. The deep sea provides a natural environmental gradient within which to study changes in different diversity metrics, but traits of deep-sea fish are not widely known, hampering the application of functional diversity to this globally important system. We used morphological traits to determine the functional richness and functional divergence of demersal fish assemblages along the continental slope in the Northeast Atlantic, at depths of 300–2,000 m. We compared these metrics to size diversity based on individual body size and species richness. Functional richness and size diversity showed similar patterns, with the highest diversity at intermediate depths; functional divergence showed the opposite pattern, with the highest values at the shallowest and deepest parts of the study site. Species richness increased with depth. The functional implications of these patterns were deduced by examining depth-related changes in morphological traits and the dominance of feeding guilds as illustrated by stable isotope analyses. The patterns in diversity and the variation in certain morphological traits can potentially be explained by changes in the relative dominance of pelagic and benthic feeding guilds. All measures of diversity examined here suggest that the deep areas of the continental slope may be equally or more diverse than assemblages just beyond the continental shelf.

## Introduction

Understanding biotic responses to environmental change remains a major challenge in ecology. Increasingly, approaches based on quantifying the functional traits of species are seen as a useful way to meet this challenge (e.g. Harfoot et al., 2014; Violle et al., 2014; Pawar, Woodward & Dell, 2015). Over the last decade, trait-based approaches have thus become central to ecology in both terrestrial and marine systems (McGill et al., 2006; Bremner, 2008; Litchman et al., 2010; Webb et al., 2010; Tyler et al., 2012; Mouillot et al., 2013). Using traits, rather than taxonomy, to describe communities confers several benefits, such as being more widely applicable to other ecosystems that may share function even with no taxonomic overlap, reducing the number of variables from hundreds of species down to only a few traits, and having a clearer connection to the function and properties of the system than do taxonomic lists (Bremner, 2008; Enquist et al., 2015; Schmitz et al., 2015). However, the extent to which we can predict the response of ecosystems to environmental change based on the traits of species remains a fundamental question in ecology (Sutherland et al., 2013), and more studies of how different dimensions of diversity vary across environmental gradients are needed.

A trait-based approach that has often been applied to marine systems, including the deep sea, uses size-based metrics (e.g. Blanchard et al., 2005; Collins et al., 2005; Piet & Jennings, 2005; Mindel et al., 2016). Body size is important in the oceans because fish grow several orders of magnitude over the course of their lives, and size, rather than species identity, often determines what prey is consumed (Cohen et al., 1993; Scharf, Juanes & Rountree, 2000; Jennings et al., 2001). Body size can be used to calculate a distinct measure of diversity, based solely on the range of individual sizes present in an assemblage, irrespective of species identity (e.g. Ye et al., 2013; Rudolf et al., 2014; Quintana et al., 2015). The diversity of individual sizes present could give more information about the range of size-based niches a fish community is occupying than does a mean or a maximum size. Leinster & Cobbold (2012) proposed a measure of diversity based on Hill numbers (Hill, 1973) that allows traditional measures of diversity based on richness and evenness to be adjusted to account for the relative similarity of the biological units of assessment. Similarity can be based on any trait, and although typically the biological units will be species, the method can be generalised to any biologically meaningful group, including size classes.

Traits other than body size also impact assemblage function. For example, gape size can be used as a proxy for what prey are consumed (Boubee & Ward, 1997) and tail measurements can be used to estimate swimming capabilities (Fisher et al., 2005). In the deep sea, variation in some morphological characteristics has been attributed to the habitat occupied. For example, species that aggregate at seamounts are deep-bodied to cope with the strong currents in these areas (Koslow, 1996; Koslow et al., 2000) and deeper-living species have more elongated body plans to increase swimming efficiency (Neat & Campbell, 2013). Locomotory capacity also declines with depth, which is likely a response to decreased light for vision that relaxes the demand for high activity levels needed to obtain prey or escape predation (Childress, 1995). Age at maturity increases with depth while fecundity and potential rate of population growth decrease (Drazen & Haedrich, 2012), ultimately having important implications for the productivity and resilience of deep-sea populations. Traits are also able to predict where or on what a species is feeding. Species that vertically migrate through the water column to feed on pelagic prey have worldwide impacts for carbon storage in the deep sea (Trueman et al., 2014). Furthermore, scavengers exhibit different traits to non-scavengers due to the high-energy reward of carrion compared to the low food availability for predators in the deep (Haedrich & Rowe, 1977; Collins et al., 2005). Large food falls are an important resource in the deep sea (Hilario et al., 2015) and scavengers that exploit this resource must possess traits such as the ability to undergo prolonged starvation, recognition of carrion odours, and sufficient motility to locate and reach the carcass (Tamburri & Barry, 1999).

Thus, traits determine what individuals feed on, where they live, and ultimately the function of deep-sea ecosystems. How communities function is not constant throughout the deep sea, as it is now known to be a diverse environment (Danovaro, Snelgrove & Tyler, 2014). The continental slope, which links shallow waters to the abyssal plain, experiences profound environmental changes due to depth, such as increased pressure and decreased temperature, light and food availability (Lalli & Parsons, 1993; Kaiser et al., 2011). These changes mean that assemblage structure varies more on a vertical gradient than it does horizontally (i.e. spatially; Angel, 1993; Lalli & Parsons, 1993; Kaiser et al., 2011), but what these structural changes mean in terms of distribution of traits and function is not yet known.

A popular approach that uses traits as building blocks is functional diversity (Tilman, 2001; Petchey & Gaston, 2002), which aims to quantify differences and similarities in function and role between species. Higher species richness may not necessarily confer a more diverse ecosystem if species overlap in the roles they perform (Walker, 1992); functional diversity aims to address this by quantifying the distinctness of different species based on biological traits. How traits are partitioned among groups of co-occurring species is debated. The ‘limiting similarity’ hypothesis predicts that species that occupy similar niches will not be able to co-exist due to interspecific competition (Macarthur & Levins, 1967), resulting in high diversity of traits (Mouillot, Dumay & Tomasini, 2007). The ‘environmental filtering’ hypothesis states that species must adapt in similar ways to local abiotic conditions, resulting in the co-existence of similar species (Keddy, 1992; Violle et al., 2007) and hence low trait diversity (Mouillot, Dumay & Tomasini, 2007). Alternatively, under the neutral hypothesis (Hubbell, 2001; Hubbell, 2005), no species are at a competitive advantage or disadvantage, so assemblages are formed by stochastic processes. Along the depth gradient of the continental slope, resource availability declines and environmental conditions become more extreme (Carney, 2005). It could therefore be expected that functional diversity will be highest in the shallowest areas where ‘limiting similarity’ causes species to occupy different niches (Macarthur & Levins, 1967), while in the deepest areas, the harsh conditions result in ‘environmental filtering’ (Keddy, 1992; Violle et al., 2007) and hence a reduction in functional diversity.

We use the depth gradient of the continental slope to compare the trait-based approaches of functional diversity calculated using species-level morphological traits, and size diversity calculated using individual body size data, to a simple measure of taxonomic diversity, species richness. We investigate the drivers behind patterns in functional diversity by examining average morphological trait values across the depth gradient. This allows us to deduce which traits are contributing most to values of functional diversity and also to explore how morphological traits relate to the dominance of feeding guilds. Feeding guilds were established using stable isotope analysis, which reveals the position of an individual in the food chain from the relative concentrations of light and heavy isotopes of nitrogen and carbon in body tissue (e.g. Michener & Schell, 1994), hence allowing us to class species as either benthic or pelagic feeders. We use this combination of taxonomic and trait-based approaches to answer for the first time in deep-sea fish assemblages the following key questions: i) How does functional diversity based on species-level traits vary along a depth gradient? ii) How do patterns in functional diversity compare to depth-dependent changes in species richness and the diversity of individual body sizes? iii) What traits are driving the changes in functional diversity along a depth gradient, and how do these traits relate to the relative dominance of feeding guilds?

## Materials and Methods

### Study site

**Figure 1:**
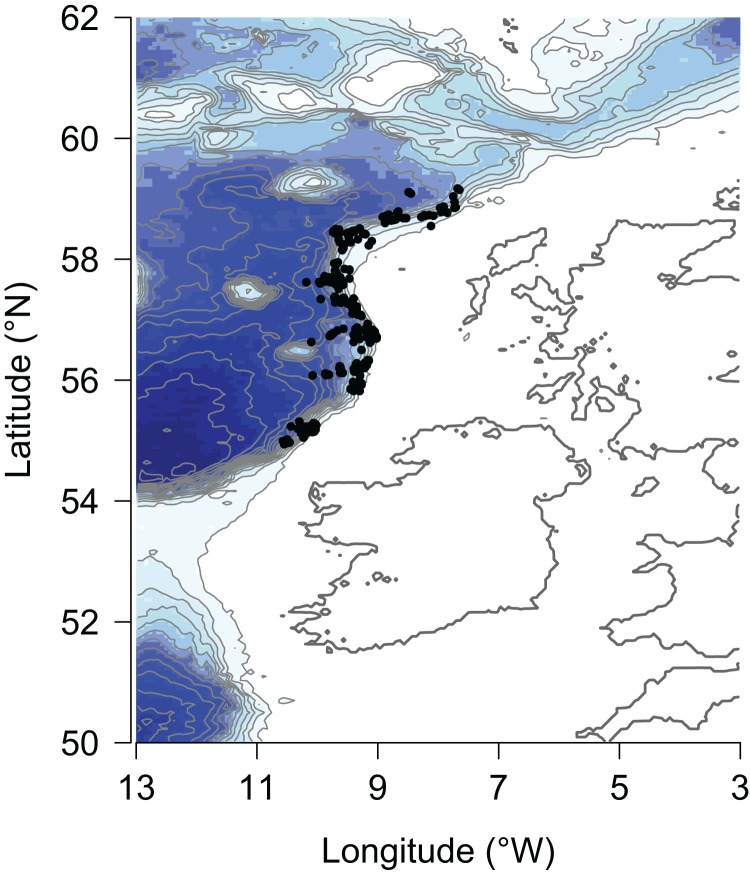
Location of hauls of the Marine Scotland deep-water bottom trawl survey along the continental slope of the Rockall Trough from 1998–2013. Shading indicates depth, with white representing the shallowest, and dark blue representing the deepest areas.

Data were collected on Marine Scotland’s deep-water bottom trawl survey of demersal fish using *MRV Scotia* in September 1998, 2000, 2002, 2004–2009 and 2011–2013 (Neat & Burns, 2010). The study area is within ICES area VIa at latitude of 55–59°N and a longitude of approximately 9°W (Fig. 1), along the continental slope of the Rockall Trough in the Northeast Atlantic, at depths of 300–2,067 m. Mesopelagic fish (those that live in the water column) and invertebrates were excluded from the analysis due to the gears being adapted to sample only demersal fish (those that live on or around the seabed, including those classified as benthopelagic).

In order to focus solely on depth-related trends, we controlled for temporal variation by pooling hauls into stations that were re-sampled through time. We then used metrics that were averaged over time within each station in all analyses. Hauls were grouped into the same station if they were in the same ICES statistical rectangle (of area 1° longitude by 30′ latitude) and within 100 m of each other in depth. The depth of the station was taken as the mean of the depths of the hauls in that station. Hauls that were not repeated across years were still included as they were assumed to occur randomly with respect to time and depth. The dataset consisted of 80 stations, including 22 stations with only one representative haul, and 58 stations where hauls were repeated over at least two years allowing for time-averaging (Table S1).

### Data collection

During the survey, catch was identified to the finest taxonomic resolution possible, which was species level for 99.9% individuals caught. The full classification of taxa was determined using the World Register of Marine Species (WoRMS Editorial Board, 2013). Photographs taken on board in 2013 were used for subsequent morphological measurements using the measuring software ImageJ (Schneider, Rasband & Eliceiri, 2012). Morphological data were collected for 31 species (Table 1) that together account for 84% of all biomass caught over the duration of the survey. These species were selected for their abundance and in order to include species that span all depths. Data for additional species could not be collected due to time constraints in photographing individuals on the survey. Measurements were replicated by using photographs of multiple individuals; the number of replicates differed among species (Table 1).

**Table 1:** Species-level trait data for the 31 species for which morphological measurements were collected. L_max_ was downloaded from FishBase (Froese & Pauly, 2016) or calculated from the survey data (please see Methods for details), feeding guild classification was based on stable isotope analyses, and all other traits were measured from photos taken on Marine Scotland’s deep-water trawl survey in 2013. Please refer to Fig. 2 and Table 2 for definitions and calculations of traits.

Species	Number of individuals measured	Relative head size (cm/cm)	Caudal fin aspect ratio (cm^2^/cm^2^/cm)	Relative eye size (cm/cm)	Eye position	Angle of mouth in relation to lateral line (°)	Relative surface area of mouth protrusion (cm^2^/cm)	Relative gape size (mm^2^/cm)	Feeding guild	L_max_ (cm)
*Alepocephalus agassizii*	22	0.29	3.11	0.074	Side	25.6	0.34	75.7	Pelagic	123.0
*Alepocephalus bairdii*	19	0.21	5.29	0.065	Side	23.3	0.19	43.8	Pelagic	127.4
*Antimora rostrata*	16	0.21	2.77	0.044	Side	16.5	0.17	58.2	Unknown	87.0
*Aphanopus carbo*	19	0.19	3.09	0.032	Side	16.9	0.00	55.8	Pelagic–high	129.0
*Apristurus aphyodes*	13	0.24	0.38	0.013	Side	9.3	0.00	20.4	Benthic	85.0
*Argentina silus*	20	0.19	3.53	0.087	Side	26.5	0.13	14.9	Pelagic	81.1
*Bathypterois dubius*	18	0.16	3.10	0.010	Side	21.1	0.17	24.8	Unknown	29.0
*Bathysaurus ferox*	6	0.14	2.19	0.018	Side	28.8	0.00	97.2	Unknown	74.2
*Beryx decadactylus*	17	0.24	3.93	0.111	Side	35.1	1.30	93.4	Unknown	100.0
*Cataetyx laticeps*	10	0.21	NA	0.020	Top	28.2	0.51	101.1	Benthic–suspension	101.0
*Centroscymnus coelolepis*	1	0.15	2.58	0.021	Side	NA	NA	35.5	Benthic	122.0
*Chimaera monstrosa*	19	0.15	NA	0.037	Side	48.3	0.08	7.9	Benthic	150.0
*Coelorinchus caelorhincus*	16	0.18	NA	0.045	Side	50.9	0.10	10.2	Benthic–low	48.0
*Coelorinchus labiatus*	20	0.26	NA	0.046	Side	57.7	0.12	5.9	Unknown	50.0
*Coryphaenoides guentheri*	21	0.17	NA	0.044	Side	35.6	0.06	6.7	Benthic	55.3
*Coryphaenoides mediterraneus*	19	0.10	NA	0.023	Side	47.5	0.19	19.8	Benthic–low	105.8
*Coryphaenoides rupestris*	29	0.15	NA	0.038	Side	56.0	0.27	25.4	Pelagic	127.7
*Halargyreus johnsonii*	20	0.22	2.72	0.061	Side	42.7	0.22	27.9	Unknown	56.0
*Halosauropsis macrochir*	11	0.13	NA	0.012	Side	44.7	0.08	15.9	Benthic–high	90.0
*Harriotta raleighana*	9	0.30	NA	0.027	Side	NA	NA	6.6	Unknown	120.0
*Helicolenus dactylopterus*	20	0.31	2.27	0.076	Side	46.5	0.67	76.3	Benthic	47.0
*Hoplostethus atlanticus*	15	0.30	3.82	0.067	Side	40.2	0.82	140.8	Benthic	75.0
*Hydrolagus affinis*	9	0.20	0.49	0.029	Side	NA	NA	19.3	Unknown	131.6
*Lepidion eques*	20	0.20	0.92	0.061	Middle	42.9	0.16	27.0	Benthic–low	44.0
*Merluccius merluccius*	6	0.20	1.41	0.036	Middle	26.7	0.50	85.0	Benthic–high	140.0
*Mora moro*	20	0.18	1.89	0.064	Middle	32.6	0.37	66.8	Unknown	80.0
*Nezumia aequalis*	17	0.16	NA	0.057	Middle	31.0	0.06	7.6	Benthic–high	37.8
*Phycis blennoides*	20	0.19	1.33	0.048	Mixed	33.6	0.55	49.8	Benthic	110.0
*Spectrunculus grandis*	14	0.16	NA	0.024	Mixed	32.9	0.24	30.5	Unknown	147.3
*Synaphobranchus kaupii*	20	0.11	NA	0.013	Side	50.4	0.00	9.6	Benthic–low	100.0
*Trachyrincus murrayi*	20	0.20	NA	0.036	Side	54.1	0.15	17.7	Benthic–low	66.7

**Figure 2:**
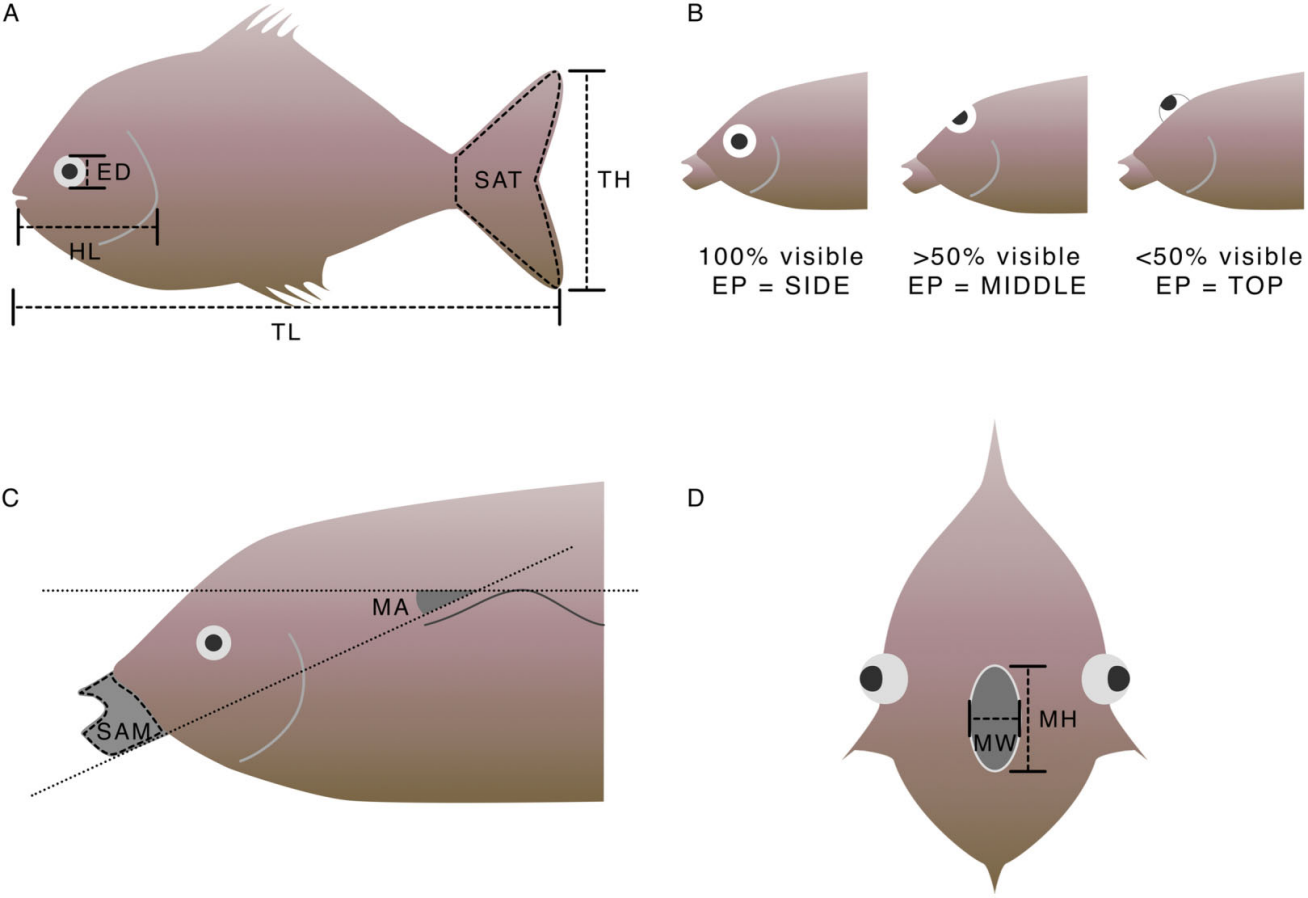
How morphological measurements were taken using photographs (panels A, B and C) and on board the survey (panel D). Morphological traits were calculated from these measurements using the formulae in Table 2. (A) TL, total length; HL, head length; ED, eye diameter; SAT, surface area of tail; TH, tail height. (B) EP, eye position. (C) SAM, surface area of mouth protrusion; MA, mouth angle. (D) MW, mouth width; MH, mouth height.

The morphological measurements taken using photographs were total length, head length, tail height, tail surface area, eye size, eye position, angle of mouth in relation to lateral line and surface area of mouth protrusion if present (Table 2; Figs. 2A–2C). Mouth height and mouth width were measured on board due to the difficulty of photographing the mouth. Gape size was then calculated as the area of an oval with mouth height and mouth width as the diameters (Table 2; Fig. 2D). The tail measurements were used to calculate the aspect ratio of the fish, which can be used to deduce activity levels (Table 2; Fig. 2A; Pauly, 1989). Head, eye, surface area of mouth protrusion and gape size were divided by total length in order to calculate relative trait values (Table 2). Relative traits were used in all analyses because body size varies substantially within species. By controlling for body size, the relative trait value can be assumed to be constant throughout an individual’s life because it represents an inherent body plan. Relative traits represent differences in function between species regardless of body size (Table 2 and references therein). The individual correlations between each of the continuous traits can be found in Fig. S1.

**Table 2:** The morphological traits used in the calculation of functional diversity, how they were calculated from the measurements depicted in Fig. 2, and their predicted link to function.

Morphological trait	Calculation	Figure 2 panel	Function/Strategy	Reference
Relative head size	${{HL} \over {TL}}$	A	Approach to prey; use of space	Reecht et al. (2013)
Caudal fin aspect ratio	$\left({{{T{H^2}} \over {SAT}}} \right)/TL$	A	Swimming speed; correlates with life history and physiological characteristics	Pauly (1989) and Fisher et al. (2005)
Relative eye size	${{ED} \over {TL}}$	A	Visual sensitivity and/or acuity	Sibbing & Nagelkerke (2001)
Eye position	*EP*	B	Vertical position in the water column	Clavel et al. (2013)
Angle of mouth in relation to lateral line	*MA*	C	Prey capture mode; vertical position in the water column of prey	Piet (1998) and Sibbing & Nagelkerke (2001)
Relative surface area of mouth protrusion	${{SAM} \over {TL}}$	C	Prey capture mode and speed	Sibbing & Nagelkerke (2001)
Relative gape size	$\left({\pi {{MH*MW} \over 2}} \right)/TL$	D	Size of prey targeted	Boubee & Ward (1997)
L_max_	See Methods	NA	Correlates with size at maturity, fecundity, growth rate and longevity	Winemiller & Rose (1992) and Froese & Binohlan (2000)

Total length was measured on board the survey for all individuals caught, in addition to the measures taken using the photographs of subsets of individuals. For 12 (39%) of the 31 species for which morphological measurements were taken (hence the species on which most analyses presented here focus), it was inappropriate to measure total length due to tails commonly breaking off in the net, so alternative measurements were taken and converted to total length using conversion factors calculated from a subset of the data (Table S2).

Subsets of the survey data were used to calculate conversion factors for translating the total length measurements to weight. Predicted weights were then standardised by controlling for the duration of time spent trawling. This measure of biomass caught per hour of trawling was used in all further analyses as the measure of abundance.

The species-level measure of body size to be included in the calculation of functional diversity was the maximum recorded length of a species, or L_max_. L_max_ was set as the maximum length listed on FishBase (Froese & Pauly, 2016) or the maximum length recorded on the survey, whichever was the greater (Mindel et al., 2016). Of the 31 species for which morphological data were available, one (*Apristurus aphyodes*) did not have an L_max_ listed on FishBase. Therefore, its L_max_ was set as that of the largest species of that genus caught on the survey (*Apristurus manis*). Standard Lengths on FishBase were converted to total length using conversion factors calculated from the survey data where possible (Table S2). If there was no survey-derived conversion factor available (due to total length being measured on the survey and Standard Length being provided by FishBase) then the conversion factor listed on FishBase was used. Where both conversion factors were missing, we used an average conversion factor that was calculated across all species caught on the survey for which there was a Standard Length conversion available.

Stable isotope data were available for 21 of the species for which morphological data were collected. The stable isotope analyses are described in Trueman et al. (2014; data are available at Dryad Digital Respository doi: 10.5061/dryad.n576n). The isotopic dataset was compared to a meta-dataset of diet studies based on stomach content analyses (Trueman et al., 2014). Where species were present in both datasets, stable isotope compositions clearly distinguished between species categorised as feeding on either benthic (seabed) or pelagic (water column) prey (Trueman et al., 2014). Stable isotope compositions were subsequently used to assign feeding guild to species and individuals lacking reliable stomach content data (Trueman et al., 2014). The distinction between benthic and pelagic feeders was less pronounced in the assemblage at 500 m, as the diets of the two guilds are similar at this depth. However, species could still be assigned to a feeding guild based on their relative isotope signatures throughout the rest of their depth range. Specialised signatures within these two feeding guilds could be established in some cases: if the smallest individual sampled for that species was in the upper half of stable isotope space for that category, the species was defined as high trophic level; if the largest individual sampled was in the lower half of stable isotope space, the species was defined as low trophic level; fish that feed on benthic suspension feeding prey have a noticeably enriched isotope signature for a given body size, depth and feeding guild, so were categorised separately.

### Data analysis

**Figure 3:**
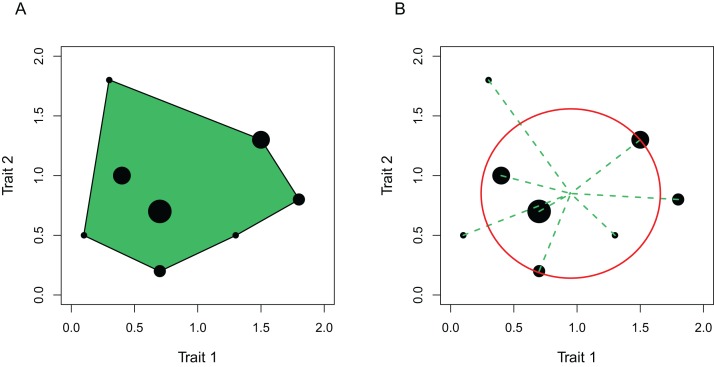
Toy example using only two traits of the calculation of (A) functional richness and (B) functional divergence. Each black point represents a species that exhibits trait values indicated by their positioning within the axes, and the size of the point represents the abundance of that species. (A) Functional richness is represented by the green shaded area, corresponding to the volume of trait space occupied by the species. (B) Functional divergence is determined by species abundances, how far those species are from the centre of gravity as determined by the species traits (illustrated by dashed lines), and how this distance compares to the mean distance to the centre of gravity (illustrated by the circle). Figures are adapted from Villéger, Mason & Mouillot (2008).

Diversity was calculated in four ways: 1) functional richness, 2) functional divergence, 3) size diversity, and 4) species richness. The two measures of functional diversity are described by Villéger, Mason & Mouillot (2008) and were calculated using the R (R Core Team, 2015) package *FD* (Laliberté & Shipley, 2011). Functional richness is an estimate of the degree to which the assemblage fills functional space (Fig. 3A; Villéger, Mason & Mouillot, 2008) and functional divergence measures how abundance is distributed within the volume of functional trait space occupied by species (Fig. 3B; Villéger, Mason & Mouillot, 2008). The traits included in the calculation of functional diversity were relative head size, aspect ratio of the caudal fin, relative eye size, eye position, angle of mouth in relation to lateral line, relative surface area of mouth protrusion if present, relative gape size, and L_max_ (Table 2). A species-level mean was calculated from the relative trait values for all continuous traits (Table 1). Functional richness does not include species abundances in its calculation; functional divergence includes a weighting of traits by species abundance, which in this case was biomass caught per hour of trawling. Due to only having trait data for a maximum of 31 species, functional diversity was only calculated using those species and their biomasses, and the rarer species were not considered. As these 31 species accounted for 84% of all biomass caught on the survey and spanned the entirety of the depth range studied, they were considered to be a good representation of the study system.

Size diversity was calculated using the generalised measure of diversity proposed by Leinster & Cobbold (2012). In this index, abundance of biologically meaningful groups and similarities between them are accounted for. Here the groups were size classes each of 10 cm in width, and abundance was calculated as the proportional biomass per hour that each size class accounts for in each station, when only the species for which morphological data were known were included. The Euclidean distance matrix (*d*) between the mid-points of size classes was converted to similarities using the formula suggested by Leinster & Cobbold (2012):

$$Similarity = {e \over {\log \left(2 \right)*d}}$$

The final input for the Leinster-Cobbold measure of diversity is the sensitivity parameter, *q*, which determines how much emphasis is given to rare species (or in this case, size classes; Leinster & Cobbold, 2012). Here a value of *q* = 1.1 was used in order to balance the richness (lower *q*) and evenness (higher *q*) components of diversity, and to be comparable to the widely used Shannon index (Shannon, 1948; Leinster & Cobbold, 2012).

Species richness was calculated using only hauls that were of 120 ± 5 min in duration in order to control for sampling effort. For this subset of hauls, the number of species present was averaged across hauls in each station. All species were included in the calculation of species richness, not just those with morphological data available. This is because calculating species richness using only the morphological subset would merely be a count of the number of species for which morphological data were available and not be meaningful in a diversity context.

The four diversity measures were calculated for each station and then analysed with respect to the depth of that station with Generalised Additive Models (GAMs) using the R (R Core Team, 2015) package *mgcv* (Wood, 2011). A smoother function of depth was used as the predictor, and the values for the test statistic, significance, R-squared, and effective degrees of freedom (e.d.f.; the flexibility of the fitted model; Wood, 2006) were extracted from the model summary.

Abundance-weighted station means were calculated for each continuous morphological trait included in the functional diversity metric and analysed with respect to the depth of the station using GAMs. The weighted mean was said to be the mean value across species, where values were weighted by the biomass caught per hour of trawling for each species. The mean observed size of individuals, irrespective of species identity, was also calculated for comparison. This value was not included in the functional diversity metric because a species-level measure of body size (L_max_) was needed. The station mean body size was therefore calculated as the average length across individuals in a station, when only individuals of species for which morphological data were obtained were included, in order to be comparable to the measures of functional and size diversity. The standard deviation of each continuous trait at each station was also calculated and analysed with respect to depth using GAMs in order to relate variation in traits to patterns seen in functional diversity. The Pearson’s product-moment correlation coefficient was calculated for the relationships between the means and standard deviations of each of the traits, and each measure of diversity.

The isotopic feeding guild data (Table 1) were interpreted using the percentage of biomass that each guild accounted for in depth bands of 200 m in width. The percentage was calculated as a proportion of the biomass accounted for by the species for which there were morphological data.

All data manipulation and analysis was performed using R version 3.1.2 (R Core Team, 2015) and figures were produced using the packages *ggplot2* (Wickham, 2009), *gridExtra* (Auguie, 2016) and *marmap* (Pante & Simon-Bouhet, 2013).

## Results

**Figure 4:**
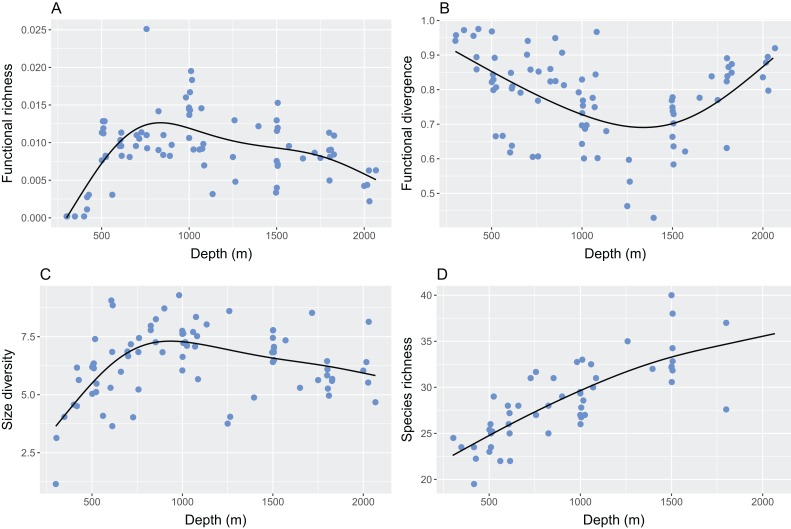
The relationship between depth and (A) functional richness, (B) functional divergence, (C) size diversity and (D) species richness. Curves represent the fitted Generalised Additive Model.

Functional richness was low in the shallowest and deepest depths, and high at around 800 m (Fig. 4A; GAM: F = 14.1, e.d.f. = 3.8, R^2^ = 0.40, p < 0.001). Functional divergence was high at both the shallow and deep ends of the depth gradient, with lowest values at around 1,300 m (Fig. 4B; GAM: F = 10.4, e.d.f. = 2.9, R^2^ = 0.31, p < 0.001). Size diversity increased to a peak at roughly 900 m, then declined as depth increased further, but remained higher in the deepest areas than in the shallowest ones (Fig. 4C; GAM: F = 10.8, e.d.f. = 3.5, R^2^ = 0.33, p < 0.001). Species richness increased significantly with depth (Fig. 4D; GAM: F = 34.2, e.d.f. = 1.9, R^2^ = 0.61, p < 0.001).

**Figure 5:**
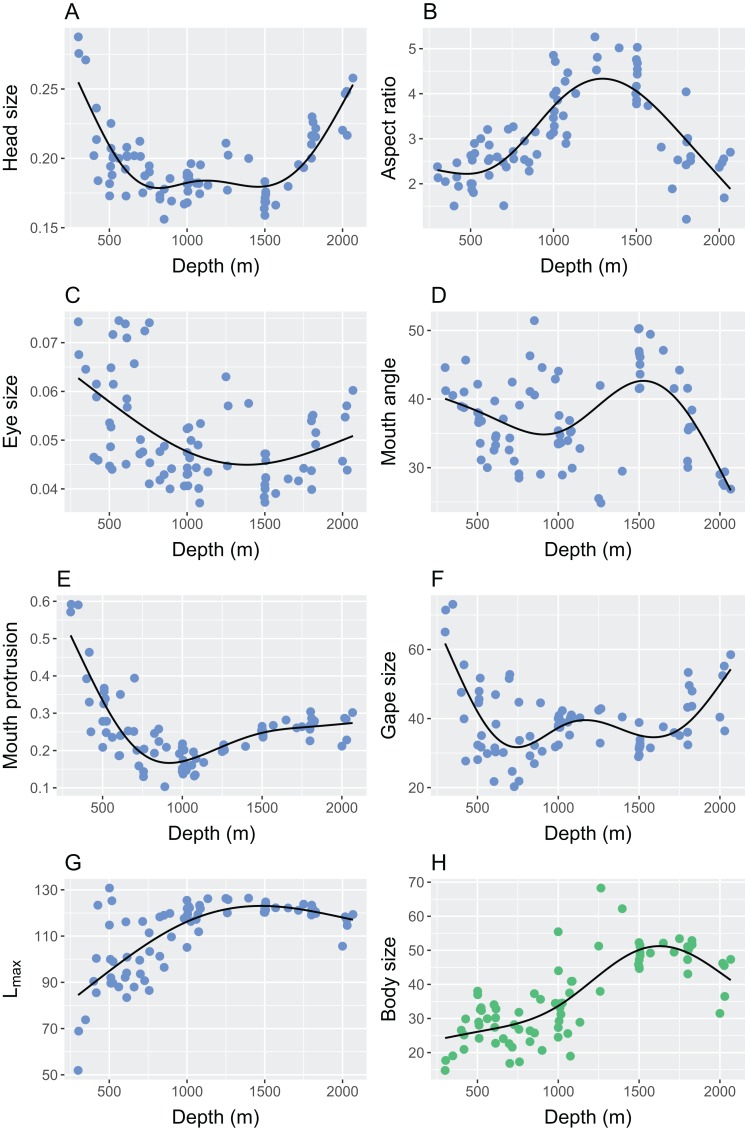
The relationship between depth and the abundance-weighted station means of continuous morphological traits. Traits that were used in the calculation of functional diversity are represented by blue points and station mean body size is represented by green points. The traits were (A) relative head size (cm/cm); (B) caudal fin aspect ratio (cm^2^/cm^2^/cm); (C) relative eye size (cm/cm); (D) angle of mouth in relation to lateral line (°); (E) relative surface area of mouth protrusion (cm^2^/cm); (F) relative gape size (mm^2^/cm); (G) L_max_ (cm); (H) body size measured as total length (cm). Please see Fig. 2 and Table 2 for definitions and calculations of traits.

**Figure 6:**
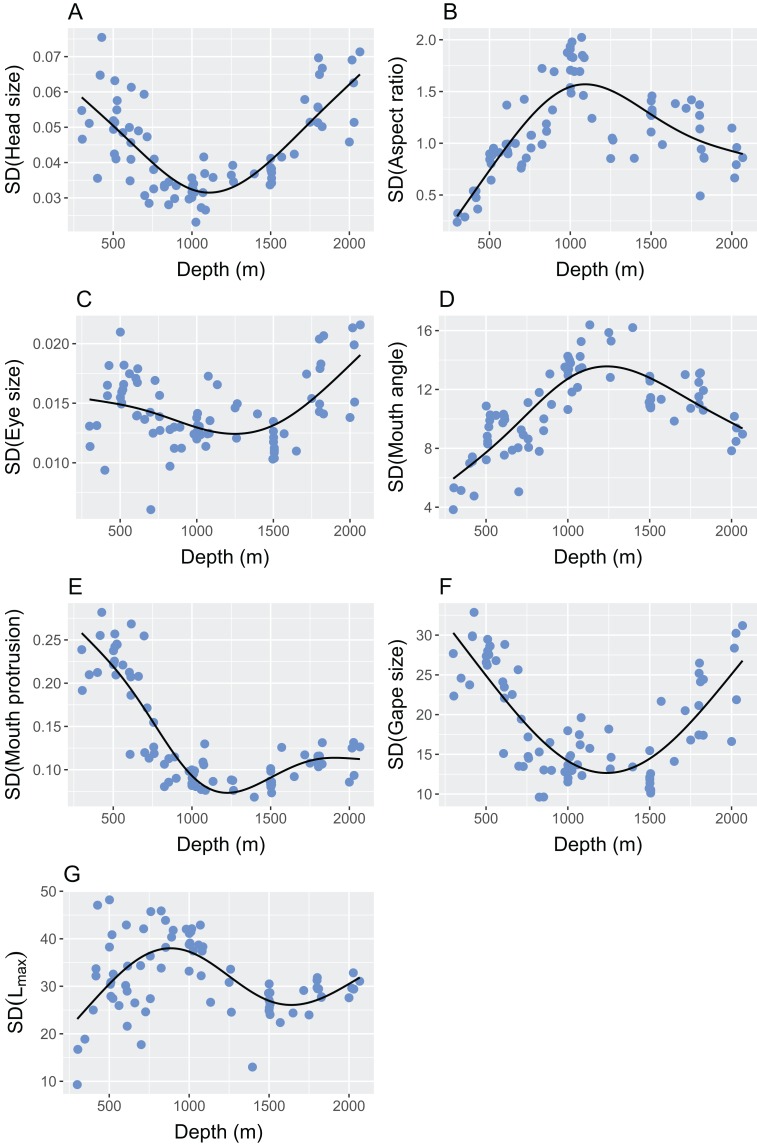
The relationship between depth and the standard deviation of the continuous morphological traits used in the calculation of functional diversity. The traits were (A) relative head size (cm/cm); (B) caudal fin aspect ratio (cm^2^/cm^2^/cm); (C) relative eye size (cm/cm); (D) angle of mouth in relation to lateral line (°); (E) relative surface area of mouth protrusion (cm^2^/cm); (F) relative gape size (mm^2^/cm); (G) L_max_ (cm). Please see Fig. 2 and Table 2 for definitions and calculations of traits.

Abundance-weighted station means and the standard deviation of continuous morphological variables changed with depth and all statistics are reported in Table 3. The mean and standard deviation of relative head size exhibited strong relationships with depth (Table 3), where heads were larger in proportion to body size, and more varied, in the shallowest and deepest parts of the study site (Figs. 5A and 6A). Mean and standard deviation of aspect ratio also varied strongly with depth (Table 3), showing peaks at 1,000–1,500 m (Figs. 5B and 6B). Depth did not explain as much variation in relative eye size as it did relative head size (Table 3), but there was still a significant relationship and eye size was largest at the shallowest depths (Fig. 5C). Variation in eye size was highest at the deepest depths (Fig. 6C). The mean angle of the mouth in relation to the lateral line varied with depth but the variance explained was low (Table 3). However, the standard deviation of the mouth angle showed a highly significant pattern with depth (Table 3), with the highest variation at intermediate depths (Fig. 6D). The mean and standard deviation of the relative surface area of the mouth protrusion exhibited strong relationships with depth (Table 3) where both values were high in the shallows then decreased and remained constant from 1,000–2,000 m (Figs. 5E and 6E). Depth explained an intermediate amount of variation in mean relative gape size (Table 3), which showed a pattern similar to that of head size, with the highest values at the shallowest and deepest parts of the study site (Fig. 5F). The standard deviation of relative gape size showed a similar pattern with depth (Fig. 6F), but the variance explained was much higher than for mean gape size (Table 3). The mean of L_max_ increased to approximately 1,000 m then remained high (Table 3; Fig. 5G). Depth explained less variation in the standard deviation of L_max_ than its mean (Table 3) but it did show a peak at around 800 m (Fig. 6G). Station mean body size, which was calculated at the individual level rather than by weighting by species abundances, increased up to 1,500 m and then declined (Table 3; Fig. 5H).

**Table 3:** Statistics extracted from Generalised Additive Models (GAMs) on the relationships between trait means and trait variances in a station, and the depth of that station.

Trait	Calculation	F	e.d.f.	R^2^	p
Relative head size (cm/cm)	Mean	37.6	3.9	0.65	< 0.001
	SD	33.6	3.3	0.62	< 0.001
Caudal fin aspect ratio (cm^2^/cm^2^/cm)	Mean	34.7	3.8	0.63	< 0.001
	SD	36.7	3.4	0.64	< 0.001
Relative eye size (cm/cm)	Mean	10.5	2.3	0.28	< 0.001
	SD	9.4	2.9	0.29	< 0.001
Mouth angle (°)	Mean	7.3	3.7	0.28	< 0.001
	SD	39.0	3.4	0.65	< 0.001
Relative surface area of mouth protrusion (cm^2^/cm)	Mean	46.6	3.8	0.70	< 0.001
	SD	81.4	3.8	0.80	< 0.001
Relative gape size (mm^2^/cm)	Mean	14.9	3.9	0.42	< 0.001
	SD	40.1	3.0	0.65	< 0.001
L_max_ (cm)	Mean	31.8	2.5	0.56	0.004
	SD	10.7	3.5	0.34	< 0.001
Individual body size (cm)	Mean	35.4	3.6	0.64	< 0.001

**Note:**

e.d.f., effective degrees of freedom; the flexibility of the fitted model (Wood, 2006). Please refer to Fig. 2 and Table 2 for calculations and definitions of traits.

Pearson’s product-moment correlation coefficients are reported for all diversity metrics and traits in Table S3. Functional richness was particularly correlated with the standard deviation of the aspect ratio (R = 0.54) and the standard deviation of the angle of mouth in relation to lateral line (R = 0.57). Functional divergence was particularly correlated with the standard deviation of the surface area of the mouth protrusion (R = 0.54), standard deviation of relative gape size (R = 0.51), and mean of the mouth protrusion (R = 0.53). Size diversity was particularly correlated with standard deviation of aspect ratio (R = 0.56), standard deviation of L_max_ (R = 0.54) and mean of L_max_ (R = 0.52). Functional richness was associated with size diversity (R = 0.42) and inversely related to functional divergence (R = −0.38).

**Figure 7:**
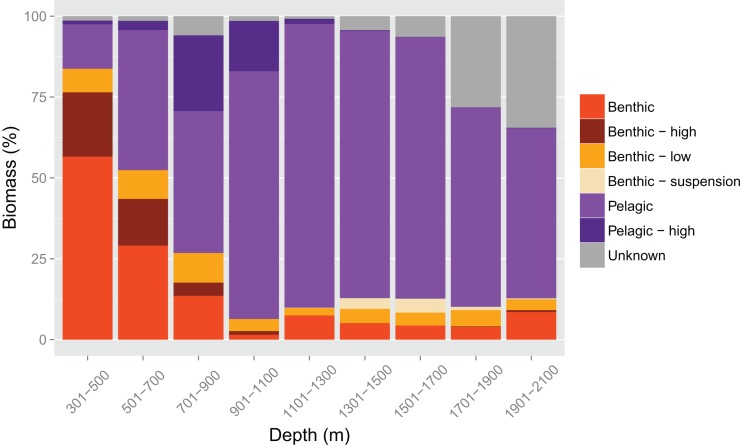
Biomass accounted for by each feeding guild in 200 m depth bands, as a percentage of the biomass accounted for by species for which morphological data were known. ‘Benthic’ refers to species feeding in the benthic environment. If they could be assigned specifically to high or low trophic levels (see Methods for details) then they are listed as ‘Benthic–high’ or ‘Benthic–low’ accordingly. Species that feed on benthic suspension feeders exhibit a distinct isotope signature so are presented as ‘Benthic–suspension.’ ‘Pelagic’ refers to species feeding in the pelagic environment and ‘Pelagic–high’ represents those that could be distinguished as high trophic level.

Relative contributions of feeding guilds to total biomass changed with depth (Fig. 7). Benthic feeders were the largest component of biomass up to 700 m then declined as depth increased. The benthic feeders that were of particularly high or low trophic levels followed the same pattern, but those of a high trophic level virtually disappeared at around 1,100 m. The specialised fish that feed on benthic suspension feeders lived mainly at 1,300–1,900 m. Generalist pelagic feeders increased with depth and dominated the biomass from 700 m, then started to decline in dominance at particularly deep depths. The high trophic level pelagic feeders were abundant only from 500–1,100 m. The biomass accounted for by species for which isotopic signatures were not known increased with depth.

## Discussion

The four measures of diversity examined here exhibit different patterns along a depth gradient, but all show intermediate or high values in the deepest part of the study site (Fig. 4). At the shallower end of the continental slope, functional richness, size diversity and species richness indicate low levels of diversity, but functional divergence is high. This implies that species are widely and unevenly distributed around the small amount of trait-space occupied (Fig. 3). The deepest areas exhibit similar patterns but functional richness and size diversity are higher than in the shallowest areas. Functional richness and size diversity are both highest at around 800–1,000 m in depth where functional divergence is low, implying that species are evenly distributed around a wide range of trait space. The conflicting patterns of the two different measures of functional diversity have been found previously in a global analysis of reef fish communities, and it has been suggested that including the abundance of species in the calculation of functional diversity (as is the case for functional divergence, but not functional richness) is particularly important in understanding patterns (Stuart-Smith et al., 2013). The similarities between functional richness and size diversity are striking as they are calculated using completely different information: functional richness uses species-level traits that have been controlled for body size, and size diversity uses variation in individual body sizes. That they correlate highly could imply that size-based metrics capture much of the information that is conveyed by species-level functional traits. Species richness increases with depth up to 1,500 m; data beyond this depth are missing due to the reduced dataset of hauls that were equal in duration not spanning the entire depth range.

The low values of functional richness, size diversity and species richness at the shallowest areas of the study site is surprising as it is often thought that diversity is higher in shallower waters where primary production is higher and there is more variation in environmental conditions (Price, Keeling & O’Callaghan, 1999; Ellingsen, 2002; Zintzen et al., 2011). Conversely, the deep sea has only been colonised recently in terms of geological time and there is a global decrease in species richness up to 8,000 m (Priede & Froese, 2013). However, alternative patterns of species richness have been postulated in the deep sea. A unimodal relationship where species richness is highest between 1,000–3,000 m has been found (Priede et al., 2010; Brown & Thatje, 2014) and the increase in species richness seen in our study could be consistent with this pattern if depths beyond the study site were to be sampled.

The high species richness, high functional divergence, and intermediate to high values of size diversity seen in the deepest part of this study site can be explained in three ways. Firstly, biodiversity and functional diversity are influenced by the range and quality of food sources, as well as total productivity (Gambi et al., 2014), implying that even if quantity of resources is lower in the deep (Carney, 2005), functional divergence could still be high if there is a heterogeneous food supply. This is consistent with the ‘limiting similarity’ hypothesis (Macarthur & Levins, 1967) whereby high trait diversity results from interspecific competition preventing species from occupying similar niches. In contrast, the declining functional richness at depth supports the ‘environmental filtering’ hypothesis (Keddy, 1992; Violle et al., 2007), perhaps because species share similar traits to cope with the extreme environmental conditions. Secondly, fishing in this study region mostly occurs above 1,200 m in depth so the deepest fish assemblages are not harvested. Human exploitation has been known to decrease diversity (de Boer & Prins, 2002; Tittensor et al., 2007; Nañola, Aliño & Carpenter, 2011), which may explain the high diversity in areas outside of human impacts. Fishing pressure is also likely to have supressed size diversity in the shallower areas due to its well-known impact on body sizes (Bianchi et al., 2000). Thirdly, it is hypothesised that the peak in species richness generally found at 1,000–3,000 m is due to a peak in speciation rates that occurs at the physiological boundary where shallow-living species became adapted to the low temperature and high pressure beyond these depths (Brown & Thatje, 2014).

The patterns in functional and size diversity can also be examined within the context of the distribution of individual functional traits (Fig. 5). Body size is known to be a particularly important functional trait in marine species (Winemiller & Rose, 1992; Froese & Binohlan, 2000; Cohen et al., 1993; Scharf, Juanes & Rountree, 2000; Jennings et al., 2001), but as most other continuous morphological traits were calculated relative to body size, their individual relationships with depth illustrate that functional traits of an assemblage are not solely determined by body size. For example, in the shallowest areas, observed body size is small while relative gape size is high. This means that for their size, the species that occupy shallower depths will have relatively larger gapes, even if this does not necessarily equate to them having the largest observed gape of all individuals in the study system.

The links between trait values and function can be observed by using stable isotope data to illustrate the relative dominance of different feeding guilds across depths (Fig. 7). Benthic feeders dominate in the shallowest areas of the slope and then drop off sharply to remain low in abundance from 900 m. This pattern mirrors that shown by the relative surface area of the mouth protrusion, which is used to suck up prey from the benthos. Similarly, the dominance of pelagic feeders at 900–1,700 m (in this study: the greater argentine, *Argentina silus*; Agassiz’s slickhead, *Alepocephalus agassizii*; Baird’s slickhead, *Alepocephalus bairdii*; the roundnose grenadier, *Coryphaenoides rupestris*; the black scabbardfish, *Aphanopus carbo*) is mirrored by several traits. The high aspect ratios of the caudal fin at these depths equate to increased swimming capabilities and are common in species that live or feed in the pelagic ocean (Pauly, 1989; Sambilay, 1990). These species also have small heads and gapes in relation to their body length (Fig. 5). As they feed mainly on planktonic invertebrates (Froese & Pauly, 2016), aside from the black scabbardfish, which is a top predator, it is unnecessary for them to have large mouths. The weighted mean head and gape size are highest in the shallowest and deepest areas (Figs. 5A and 5F), where a wider range of prey sources are utilised (Fig. 7).

Variation in traits generally mirror the patterns seen in the means of those traits (Fig. 6), aside from relative gape size and angle of mouth in relation to lateral line, which show lower correlations between the mean and the standard deviation than in other traits (Table S3). Despite the low correlation for relative gape size, both the mean and standard deviation are highest at the shallow and deep areas of the study site. Mouth angle shows an inconclusive relationship with depth when the mean is used, but a very strong relationship when the standard deviation is used. The high variation at intermediate depths is perhaps more informative than the mean because it could be explained by the dominance of pelagic feeders in a similar way to the aforementioned traits. It may be that there is no particular mouth angle that is selected for in pelagic feeders, hence species show a wider range of angles. In comparison, the shallowest and deepest areas exhibit lower variation because a certain mouth angle is selected for in the benthic feeders in the shallows, and potentially in the unknown feeders in the deep (Fig. 7).

Depth-dependent patterns in the variation of all traits can be linked to diversity metrics, apart from relative head size and eye size, which show lower correlations with diversity (Table S3). The mean and variation in L_max_ are linked to patterns in size diversity, implying that variation in observed individual sizes is at least partially explained by the potential maximum sizes of the species present. It may therefore be possible to use this species-level measure of potential size as a proxy for variation in observed sizes in data-poor scenarios. Functional divergence is particularly associated with relative gape size and the surface area of the mouth protrusion, and aspect ratio of the caudal fin links highly with functional richness and size diversity. Higher aspect ratios have been found to associate with depth generalist species in coral reefs (Bridge et al., 2016), further highlighting this trait’s role in many aspects of community assembly, and the aforementioned potential link between high aspect ratios and the dominance of pelagic feeders over a wide depth range.

The dominance of pelagic feeders at intermediate depths is mainly due to the presence of a community of Diel Vertical Migrators (DVM; Mauchline & Gordon, 1991; Trueman et al., 2014), otherwise known as the deep scattering layer. This is a mesopelagic community containing fish, invertebrates and zooplankton, which has recently been found to be particularly important for global biogeochemical cycles and carbon storage in the oceans (Irigoien et al., 2014; Trueman et al., 2014). Its relative positioning could potentially explain the patterns that we see in the two measures of functional diversity examined here. At the shallow end of the continental slope (< 500 m), the DVM community is close to the seabed, so both benthic- and pelagic-feeding demersal fish are able to exploit it. This could be consistent with the low functional richness that we see in this area, if all species are occupying similar functional space in order to exploit the same resources, and the high functional divergence, because multiple species could co-exist through fine partitioning of resources in-line with the ‘limiting similarity’ hypothesis (Macarthur & Levins, 1967). With increasing depth, the distance of the DVM community from the seabed also increases, meaning that benthic feeders are no longer able to exploit it (1,000–1,500 m). The dominance of pelagic-feeders here, and the related traits of those species, may therefore be caused by their competitive release. The low functional divergence seen at these depths may be due to the dominance of only a few species, all with similar traits that are adapted to feeding on pelagic prey, such as small gapes and high aspect ratio as discussed above. The co-existence of species with similar traits, exploiting similar resources, may be maintained by an assembly rule termed ‘emergent neutrality’ (Scheffer & van Nes, 2006). This is when species aggregate in certain areas of a niche axis and has been supported by studies on marine phytoplankton (Vergnon, Dulvy & Freckleton, 2009), pollinators (Fort, 2014) and beetles (Scheffer et al., 2015). Beyond 1,500 m, the DVM community is too far above the seabed for even the pelagic-feeding demersal fish to reach. Less is known about the feeding habits of fish species at these depths, but the high functional divergence could illustrate a high level of specialisation and exploitation of different resources among the benthic- and pelagic-feeders.

## Conclusions

Here we have shown non-linear patterns in functional and size diversity of a deep-sea demersal fish assemblage. Functional richness and size diversity are lowest at the shallowest (< 500 m) and deepest (∼2,000 m) parts of the continental slope studied; functional divergence is the opposite, with the lowest values seen at 1,000–1,500 m. Species richness increases linearly along the depth gradient, at least up to 1,500 m. Changes in functional diversity appear to be driven by traits such as caudal fin aspect ratio and relative surface area of mouth protrusion, which can in turn be linked to the dominance of different feeding guilds along the slope. Functional richness and size diversity show similar depth-dependent patterns, despite accounting for different morphological traits. Future work could incorporate individual-level traits, rather than the species-level traits used here, and could investigate the different drivers of community assembly along the continental slope.

## Supplemental Information

10.7717/peerj.2387/supp-1 Supplemental Information 1 Concatenation of hauls into stations. Description of the reduced dataset, whereby hauls were concatenated into stations if they were repeated across years in the same ICES statistical rectangle and at depths within 100 m of each other.

10.7717/peerj.2387/supp-2 Supplemental Information 2 Species for which morphological data were available, the lengths measured, and their conversion factors. The tails of many deep-sea species are easily broken off when caught by a trawl (ICES 2012). Without its tail, an individual’s total length (tip of snout to end of tail) cannot be measured, so for 39% of the study species, standard length (tip of snout to start of tail), pre-anal fin length (tip of snout to first ray of anal fin), or pre-supra caudal fin length (tip of snout to start of supra caudal fin) is measured, depending on what species it is (ICES 2012). The measured lengths can then be multiplied by a conversion factor in order to predict the total length. Conversion factors were calculated from a subset of individuals caught on the survey for which total length was available. TL = total length; SL = standard length; PAFL = pre-anal fin length; PSCFL = pre-supra caudal fin length.

10.7717/peerj.2387/supp-3 Supplemental Information 3 The Pearson’s product-moment correlation coefficient between each measure of diversity, mean trait values, and standard deviation of trait values. Trait definitions and units are those defined throughout the main manuscript.

10.7717/peerj.2387/supp-4 Supplemental Information 4 The relationships between each of the continuous trait variables included in the calculation of functional diversity. Angle of mouth in relation to lateral line (°); relative surface area of mouth protrusion (cm^2^/cm); caudal fin aspect ratio (cm^2^/cm^2^/cm); relative eye size (cm/cm); relative head size (cm/cm); relative gape size (mm^2^/cm); L_max_ (cm). Please refer to Fig. 2 and Table 2 for definitions and calculations of traits. The statistical correlations between variables were all less than 0.7, and 15/21 correlations were less than 0.5.

## Additional Information and Declarations

### Data Deposition

The following information was supplied regarding data availability: Figshare: http://dx.doi.org/10.6084/m9.figshare.1449217.
