# Supplementary material for: Functional, size and taxonomic diversity of fish along a depth gradient in the deep sea
Source: PeerJ. 2016 Sep 15;4:e2387. doi: 10.7717/peerj.2387 (PMC5028789; doi:10.7717/peerj.2387)
Supplement: Supplemental Information 1 — Description of the reduced dataset, whereby hauls were concatenated into stations if they were repeated across years in the same ICES statistical rectangle and at depths within 100 m of each other. [file peerj-04-2387-s001.docx]

**Table S1. Concatenation of hauls into stations.** Description of the reduced dataset, whereby hauls were concatenated into stations if they were repeated across years in the same ICES statistical rectangle and at depths within 100 m of each other.

| **Station number** | **ICES statistical rectangle** | **Number of years represented** | **Mean and range of depths represented (m)** |
| --- | --- | --- | --- |
| 1 | 38D9 | 6 | 1500 (1500-1500) |
| 2 | 38D9 | 1 | 1800 (1800-1800) |
| 3 | 39D9 | 3 | 417 (400-430) |
| 4 | 39D9 | 5 | 501 (500-506) |
| 5 | 39D9 | 3 | 615 (600-630) |
| 6 | 39D9 | 2 | 757 (750-764) |
| 7 | 39D9 | 9 | 1000 (955-1050) |
| 8 | 39D9 | 1 | 1264 (1264-1264) |
| 9 | 39D9 | 1 | 1396 (1396-1396) |
| 10 | 39D9 | 3 | 1506 (1500-1518) |
| 11 | 39D9 | 3 | 1828 (1800-1884) |
| 12 | 39D9 | 1 | 2067 (2067-2067) |
| 13 | 40E0 | 4 | 427 (400-450) |
| 14 | 40E0 | 4 | 728 (700-750) |
| 15 | 40E0 | 4 | 982 (950-1000) |
| 16 | 41D9 | 1 | 2027 (2027-2027) |
| 17 | 41E0 | 10 | 502 (487-528) |
| 18 | 41E0 | 1* | 715 (700-730) |
| 19 | 41E0 | 4 | 853 (809-890) |
| 20 | 41E0 | 10 | 1011 (966-1050) |
| 21 | 41E0 | 1 | 1070 (1070-1070) |
| 22 | 41E0 | 10 | 1506 (1497-1530) |
| 23 | 41E0 | 1 | 1750 (1750-1750) |
| 24 | 41E0 | 3 | 1827 (1800-1850) |
| 25 | 42D9 | 1 | 2017 (2017-2017) |
| 26 | 42E0 | 10 | 517 (500-550) |
| 27 | 42E0 | 2 | 607 (592-622) |
| 28 | 42E0 | 4 | 757 (709-800) |
| 29 | 42E0 | 1 | 890 (890-890) |
| 30 | 42E0 | 11 | 1016 (1000-1093) |
| 31 | 42E0 | 2 | 1259 (1237-1300) |
| 32 | 42E0 | 11 | 1501 (1500-1508) |
| 33 | 42E0 | 7 | 1800 (1800-1800) |
| 34 | 43E0 | 3 | 417 (409-425) |
| 35 | 43E0 | 7 | 510 (496-525) |
| 36 | 43E0 | 2 | 561 (552-570) |
| 37 | 43E0 | 1 | 696 (696-696) |
| 38 | 43E0 | 2 | 760 (750-770) |
| 39 | 43E0 | 2 | 825 (800-850) |
| 40 | 43E0 | 8 | 999 (990-1005) |
| 41 | 43E0 | 1 | 1075 (1075-1075) |
| 42 | 43E0 | 8 | 1500 (1500-1500) |
| 43 | 43E0 | 1 | 1570 (1570-1570) |
| 44 | 43E0 | 1 | 1804 (1804-1804) |
| 45 | 43E0 | 1 | 2030 (2030-2030) |
| 46 | 44D9 | 1 | 2000 (2000-2000) |
| 47 | 44E0 | 3 | 303 (300-310) |
| 48 | 44E0 | 1 | 400 (400-400) |
| 49 | 44E0 | 6 | 507 (500-540) |
| 50 | 44E0 | 8 | 612 (580-650) |
| 51 | 44E0 | 2 | 700 (700-700) |
| 52 | 44E0 | 4 | 825 (800-850) |
| 53 | 44E0 | 2 | 900 (900-900) |
| 54 | 44E0 | 9 | 1005 (994-1050) |
| 55 | 44E0 | 1 | 1081 (1081-1081) |
| 56 | 44E0 | 2 | 1250 (1250-1250) |
| 57 | 44E0 | 11 | 1506 (1500-1540) |
| 58 | 44E0 | 2 | 1650 (1650-1650) |
| 59 | 44E0 | 3 | 1810 (1800-1830) |
| 60 | 45E0 | 2 | 348 (345-350) |
| 61 | 45E0 | 4 | 523 (500-550) |
| 62 | 45E0 | 8 | 612 (570-650) |
| 63 | 45E0 | 1 | 852 (852-852) |
| 64 | 45E0 | 10 | 1001 (1000-1015) |
| 65 | 45E0 | 1 | 1134 (1134-1134) |
| 66 | 45E0 | 10 | 1506 (1500-1529) |
| 67 | 45E0 | 2 | 1717 (1700-1750) |
| 68 | 45E0 | 3 | 1802 (1800-1805) |
| 69 | 46E1 | 1 | 300 (300-300) |
| 70 | 46E1 | 2 | 525 (500-550) |
| 71 | 46E1 | 4 | 603 (597-613) |
| 72 | 46E1 | 1 | 660 (660-660) |
| 73 | 46E1 | 7 | 1005 (1000-1034) |
| 74 | 46E1 | 6 | 1501 (1500-1504) |
| 75 | 46E2 | 4 | 510 (500-540) |
| 76 | 46E2 | 4 | 1000 (1000-1000) |
| 77 | 46E2 | 2 | 1059 (1058-1060) |
| 78 | 47E1 | 3 | 1496 (1489-1500) |
| 79 | 47E2 | 2 | 1025 (1000-1050) |
| 80 | 47E2 | 1 | 1086 (1086-1086) |

* This was the only station where a haul of similar depth and location was repeated in the same year, resulting in an average being taken across hauls, but only one year being represented. All other stations listed as representing only one year consisted of a single haul.
