# Supplementary material for: Functional, size and taxonomic diversity of fish along a depth gradient in the deep sea
Source: PeerJ. 2016 Sep 15;4:e2387. doi: 10.7717/peerj.2387 (PMC5028789; doi:10.7717/peerj.2387)
Supplement: Supplemental Information 2 — The tails of many deep-sea species are easily broken off when caught by a trawl (ICES 2012). Without its tail, an individual’s total length (tip of snout to end of tail) cannot be measured, so for 39% of the study species, standard length (tip of snout to start of tail), pre-anal fin length (tip of snout to first ray of anal fin), or pre-supra caudal fin length (tip of snout to start of supra caudal fin) is measured, depending on what species it is (ICES 2012). The measured lengths can then be multiplied by a conversion factor in order to predict the total length. Conversion factors were calculated from a subset of individuals caught on the survey for which total length was available. TL = total length; SL = standard length; PAFL = pre-anal fin length; PSCFL = pre-supra caudal fin length. [file peerj-04-2387-s002.docx]

**Table S2. Species for which morphological data were available, the lengths measured, and their conversion factors.** The tails of many deep-sea species are easily broken off when caught by a trawl (ICES 2012). Without its tail, an individual’s total length (tip of snout to end of tail) cannot be measured, so for 39% of the study species, standard length (tip of snout to start of tail), pre-anal fin length (tip of snout to first ray of anal fin), or pre-supra caudal fin length (tip of snout to start of supra caudal fin) is measured, depending on what species it is (ICES 2012). The measured lengths can then be multiplied by a conversion factor in order to predict the total length. Conversion factors were calculated from a subset of individuals caught on the survey for which total length was available. TL = total length; SL = standard length; PAFL = pre-anal fin length; PSCFL = pre-supra caudal fin length.

| **Species** | **Measured length** | **Conversion factor** |
| --- | --- | --- |
| Alepocephalus agassizii | SL | 1.139 |
| Alepocephalus bairdii | SL | 1.089 |
| Antimora rostrata | TL | 1 |
| Aphanopus carbo | TL | 1 |
| Apristurus aphyodes | TL | 1 |
| Argentina silus | TL | 1 |
| Bathypterois dubius | TL | 1 |
| Bathysaurus ferox | TL | 1 |
| Beryx decadactylus | TL | 1 |
| Cataetyx laticeps | TL | 1 |
| Centroscymnus coelolepis | TL | 1 |
| Chimaera monstrosa | PSCFL | 1.31 |
| Coelorinchus caelorhincus | PAFL | 2.82 |
| Coelorinchus labiatus | PAFL | 2.5 |
| Coryphaenoides guentheri | PAFL | 3.25 |
| Coryphaenoides mediterraneus | PAFL | 4.5 |
| Coryphaenoides rupestris | PAFL | 4.33 |
| Halargyreus johnsonii | TL | 1 |
| Halosauropsis macrochir | TL | 1 |
| Harriotta raleighana | PSCFL | 1.29 |
| Helicolenus dactylopterus | TL | 1 |
| Hoplostethus atlanticus | TL | 1 |
| Hydrolagus affinis | PSCFL | 1.07 |
| Lepidion eques | TL | 1 |
| Merluccius merluccius | TL | 1 |
| Mora moro | TL | 1 |
| Nezumia aequalis | PAFL | 3.78 |
| Phycis blennoides | TL | 1 |
| Spectrunculus grandis | TL | 1 |
| Synaphobranchus kaupii | TL | 1 |
| Trachyrincus murrayi | PAFL | 3.1 |

**References**

ICES. 2012. *Manual for the International Bottom Trawl Surveys*. Series of ICES Survey Protocols. SISP 1-IBTS VIII.
