# Supplementary figures and images for: Functional, size and taxonomic diversity of fish along a depth gradient in the deep sea
Source: PeerJ. 2016 Sep 15;4:e2387. doi: 10.7717/peerj.2387 (PMC5028789; doi:10.7717/peerj.2387)

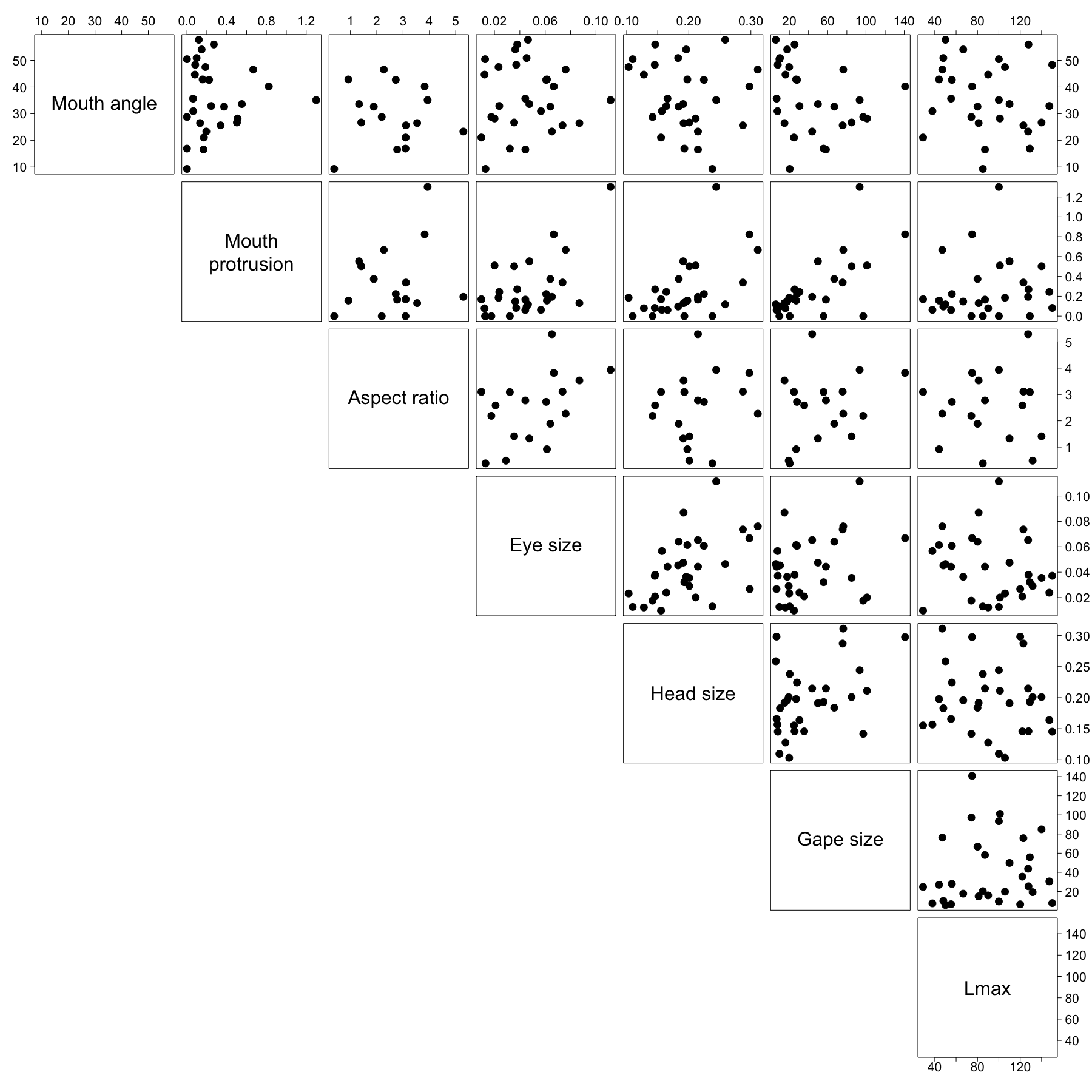

Supplement: Supplemental Information 4 — Angle of mouth in relation to lateral line (°); relative surface area of mouth protrusion (cm2/cm); caudal fin aspect ratio (cm2/cm2/cm); relative eye size (cm/cm); relative head size (cm/cm); relative gape size (mm2/cm); Lmax (cm). Please refer to Fig. 2 and Table 2 for definitions and calculations of traits. The statistical correlations between variables were all less than 0.7, and 15/21 correlations were less than 0.5. [file peerj-04-2387-s004.png]
